# Preparation and Application of Polyclonal Antibodies for the Rapid Detection of Actinidia Chlorotic Ringspot-Associated Virus

**DOI:** 10.3390/v16101600

**Published:** 2024-10-11

**Authors:** Jing Shang, Hongping Feng, Yuxuan Wang, Yunan Wang, Xiao Zhang, Zhouyu Zhang

**Affiliations:** Sichuan Engineering Research Center for Crop Strip Intercropping System and College of Agronomy, Sichuan Agricultural University, Chengdu 611130, China; hongpingfenghappy@163.com (H.F.); wangyuxuan0514g@163.com (Y.W.); 18384319857@163.com (Y.W.); aa330661@163.com (X.Z.); 18090438650@163.com (Z.Z.)

**Keywords:** AcCRaV, antibody, immunochromato graphic strip, rapid detection

## Abstract

Actinidia chlorotic ringspot-associated virus (AcCRaV, *Emaravirus actinidiae*) is prevalent in Chinese kiwifruit, leading to substantial yield reduction. The intricate nature of symptoms presents diagnostic challenges, underscoring the necessity for a rapid and accurate detection method that facilitates effective control. In this investigation, AcCRaV isolates from key kiwi-producing regions in Sichuan province were collected and analyzed, with representative strains chosen as experimental materials. Primers targeting the nucleoprotein gene of AcCRaV were designed, and their codon usage was optimized to enhance performance. Various serological methods utilizing polyclonal antibodies were developed, including ELISA, dot immunobinding assay, and AcCRaV-specific gold immunochromatographic bands (AcCRaV-GICS). Field samples exhibited high specificity and sensitivity when tested using these methods. Furthermore, the results obtained from a large number of field samples are consistent with those derived from RT-PCR analysis, further validating the applicability of our approach. A detection method capable of handling a large volume of field samples infected with AcCRaV is currently lacking; thus, our system construction provides an important reference for addressing this gap.

## 1. Introduction

The kiwifruit (*Actinidia chinensis*), renowned for its high vitamin C content [1], is cultivated on large vines. Kiwi virus disease often presents symptoms such as mottled and chlorotic leaves, leading to reduced tree vigor. Our prior study demonstrated that Actinidia chlorotic ringspot-associated virus (AcCRaV, *Emaravirus actinidiae*) had the highest detection rate in Sichuan kiwi [2]. This virus is widely distributed in various Chinese provinces including Hubei, Anhui, Shaanxi, Zhejiang, Yunnan, and Jiangxi and can infect nearly all kiwi varieties [3]. Furthermore, it frequently mixes with other viruses resulting in complex symptoms [2]. Belonging to the viral genus *Emaravirus* [4], AcCRaV induces chlorotic spots or ring spots on kiwi leaves. The virions of AcCRaV are spherical, about 80–120 nm in diameter, and are mainly distributed in the cytoplasm. AcCRaV is a negative-sense RNA virus whose genome consists of five RNA strands. The 13 nucleotides of the 5′ terminal and 3′ terminal of AcCRaV’s genome are highly conserved and reverse complementary [2]. Given the damage caused by viral diseases, precise diagnosis becomes particularly critical. Serological methods and PCR are commonly utilized methods for routine plant virus detection [5,6]. Polyclonal antibodies serve as the primary material for serological test purposes [7].

The enzyme-linked immunosorbent assay (ELISA) is widely used for detecting various viruses in barley, beans, sugarcane, hops, tobacco, potato, citrus fruits, grapes, and figs [8]. Citrus tristeza virus (CTV, *Closterovirus tristezae*) from the virus genus *Closterovirus* can be detected in young shoots and petioles using ELISA without seasonal interference [9]. Citrus psorosis virus (CPsV, *Ophiovirus citri*) from the virus genus *Ophiovirus* was successfully detected through double antibody sandwich ELISA (DAS-ELISA) and triple antibody sandwich ELISA (TAS-ELISA), with sensitivity being unaffected by the variety tested [10]. The dot-immunobinding assay (DIBA) is a simple yet highly reproducible immunodiagnostic method widely utilized in research studies [11]. Shang et al. effectively used this technique to detect Cucumber green mottle mosaic virus (CGMMV, *Tobamovirus viridimaculae*) from the virus genus *Tobamovirus*, demonstrating that DIBA exhibited four times higher than an antigen-coated plate enzyme-linked immunosorbent assay (DAC-ELISA) when applied to tissue extracts from infected plants [12]. Samsatly et al. also employed this method to detect Potato virus Y (PVY, *Potyvirus yituberosi*) from the virus genus *Potyvirus* and *Potato virus S* (PVS, *Carlavirus sigmasolani*) from the virus genus *Carlavirus* [13].

The gold immunochromatography strip (GICS), known for its high accuracy and user-friendliness, is attracting more attention in plant virology. Ren et al. (2021) demonstrated the rapid detection capability of colloidal gold strips for Soybean mosaic virus (SMV, *Potyvirus glycitessellati*) from the virus genus *Potyvirus* [5]. Niu et al. (2018) utilized a double antibody sandwich method to develop a GICS specifically targeting Tomato zonate spot virus (TZSV, *Orthotospovirus tomatozonae*) from the virus genus *Orthotospovirus*, which can be transmitted through grafting in Kiwi fruit leading to virus accumulation [14]. Despite its wide distribution, AcCRaV has received limited research attention so far [2]. Therefore, it is essential to establish an efficient and rapid detection system for AcCRaV.

In this study, we generated polyclonal antibodies against AcCRaV and validated their efficacy using ELISA and DIBA. Additionally, we developed an AcCRaV-specific gold immunochromatography strip (AcCRaV-GICS) that was satisfactorily applied for field sample detection. The colloidal gold immunochromatography test paper exhibits remarkable specificity, high sensitivity, user-friendly control, and holds significant potential for application and further development.

## 2. Materials and Methods

### 2.1. AcCRaV Isolates and Primer Design

A total of 445 kiwifruit leaf samples exhibiting symptoms such as mosaic, mottling, chlorosis, yelosis, deformity, and necrotic spots were collected from Qionglai, Pujiang, Dujiangyan, Ya’an, and Chongzhou in Sichuan Province. The results revealed that 175 out of the 445 field samples tested positive for AcCRaV across five counties with a detection rate of 39.32%. Additionally, 12 samples tested positive for Actinidia virus A (AcVA, *Vitivirus alphactinidiae*) from the virus genus *Vitivirus* at a detection rate of 2.70%, while 45 samples showed positivity for Actinidia virus B (AcVB, *Vitivirus betactinidiae*) from the virus genus *Vitivirus* at a detection rate of 10.11%. Notably, the highest detection rate was observed for AcCRaV. A polyclonal antibody was prepared based on the amino acid sequence of the nucleoprotein of AcCRaV isolate DJYI. The leaves showed obvious chlorosis and a single infection. Nucleic acid sequences were retrieved from the National Center for Biotechnology Information (NCBI), and the primers were designed using Primer Premier 5.0 software and synthesized using Biological Bioengineering (Shanghai) Technology Co., Ltd. (Shanghai, China). The primer sequences used in this experiment are provided in Supplementary Table S1.

### 2.2. Sequence and Phylogenetic Analysis

A total of 0.2 g of kiwi fruit leaf was utilized for the extraction of total RNA. For grinding, a mixture containing 0.1 mL of absolute ethanol, 0.5 mL of RNA extraction buffer, and 0.5 mL of phenol-isoamyl alcohol solution was promptly added, thoroughly mixed, and incubated on ice for 15 min. For the total RNA extraction, we followed the protocol described by Zhang et al. [15]. Subsequently, cDNA synthesis was carried out using a reverse transcription kit (Tsingke Biotechnology Co., Ltd., Beijing, China). Amplification of the fragments corresponding to the nucleoprotein gene of AcCRaV was carried out utilizing Primer STAR Max^®^ DNA Polymerase (Takara, Dalian, China) with the prepared cDNA as template material. The purified PCR products were cloned into the pMD18-T vector (Takara, Dalian, China) and subjected to sequencing analysis by Shanghai Biotechnology and Biological Engineering (Shanghai, China) Technology Co., Ltd.

The full-length nucleoprotein gene sequences of AcCRaV (abbreviated as *AcCRaV-CP*) and relevant virus isolates were aligned, and phylogenetic analysis was conducted using MEGA 7.0 software. The included sequences consisted of AcCRaV-CP-DJYI (isolate code: OQ652082), AcCRaV-CP-QL 41 (OM481013.1), AcCRaV-CP-CX16 (OM481011.1), and AcCRaV-PJ43 (OM481012.1), as well as 18 preliminary laboratory-isolated nucleoprotein gene sequences of AcCRaV obtained from Shang et al. 2022 [2], and the 41 Shaanxi isolate’s nucleoprotein gene sequences of AcCRaV from Zhao et al. 2018 [16]. A bootstrap neighbor-joining tree was generated for the phylogenetic analysis, with bootstrap values calculated based on 1000 random replications. The outgroup taxa used in this study included AmeO-COL (KJ439586.1), SOF-NAT (KJ439585.1), and isolates of European mountain ring spot virus (European mountain ash ringspot-associated virus, EMARaV), as well as AR15 (JF795554.1) and AR16 (JF795553) of the related Redbud yellow ringspot-associated virus (RYRSav).

### 2.3. Prokaryotic Expression and Purification of Recombinant Protein for Nucleoprotein of AcCRaV

After sequencing the PCR products, the AcCRaV-CP gene sequence was obtained. This sequence was codon-optimized to obtain the AcCRaV-CP’ gene sequence. AcCRaV-CP’ was obtained by adjusting the CAI value and GC content of the mRNA sequence. The whole gene sequence was synthesized by Hangzhou Hua’an Biotechnology Co., Ltd. (Hangzhou, China). The prokaryotic expression vector constructed with the optimized sequence significantly increased the soluble expression of the recombinant protein.

Specific primers, pET28a-AcCRaV-CP-F and pET28a-AcCRaV-CP-R, were designed according to the AcCRaV-CP’ genome sequence. The two BamH I and XhoI restriction endonuclease sites were arranged to the forward and reverse primers, respectively. The full-length AcCRaV-CP’ gene was amplified from the pMD18-T vector containing the AcCRaV-CP’ fragment using Primer STAR Max DNA Polymerase (Takara, Dalian, China). The PCR product was purified, digested with BamH I and XhoI endonucleases and then cloned into the prokaryotic expression vector pET28a [17].

The recombinant plasmid pET28a-AcCRaV-CP was validated through DNA sequencing and then transferred into the *Escherichia coli* expression strain Rosetta (DE3) (Tsingke Biotechnology Co., Ltd.). The *E. coli* cells containing pET28a-AcCRaV-CP were cultured in 5 mL of Luria-Bertani (LB) medium (containing 50 µg/mL of ampicillin) at 37 °C for 20 h. Then, 1 mL of the culture was inoculated into 1 L of LB medium (containing 50 µg/mL of kanamycin) at 37 °C to reach an optical density of OD600 = 0.6.

IPTG (isopropyl-beta-D-thiogalactopyranoside) was added to the bacterial culture to a final concentration of 1 mmol/L to induce the expression of the recombinant protein. The culture was then incubated at 37 °C and 160 rpm for 4 h on a shaker–incubator. Bacterial cells were collected using centrifugation at 12,000 rpm for 10 min, and the supernatant was discarded. The bacterial pellet was resuspended in 10 mL of 1 × PBS and lysed using sonication on ice at 100 W for 16 min. After centrifugation at 12,000 rpm for 10 min at 4 °C, the pellet was resuspended in 10 mL of 8 mol/L urea solution, and sonication continued at 100 W for 10 min on ice. The suspension was centrifuged at 12,000 rpm for 10 min, and the supernatant was subjected to affinity purification of His-tagged proteins in their non-denatured state. Both N- and C-terminal His-tags were retained, and Ni-NTA purification was performed using a HyPur T Ni-NTA 6FF (His-Tag) PrePacked Gravity Column (Shenggong Biological Co., Ltd., Shanghai, China).

Subsequently, the purified recombinant protein was dialyzed into 1 × PBS to eliminate imidazole. The expression and purification of AcCRaV-CP were assessed using SDS-PAGE and stained with Coomassie brilliant blue. An amount of 5 µL protein was used for SDS-PAGE and the concentration of the used gel(s) was 12.5%. The dialyzed protein was then stored in a −80 °C ultra-cold refrigerator.

### 2.4. Preparation of Horseradish Peroxidase Labeled Polyclonal Antibody

New Zealand White rabbits (two months old) were immunized subcutaneously by Hua’an Biotechnology Co., Ltd., Hangzhou, China, with 400 µg of purified recombinant AcCRaV-CP fusion protein emulsified with 400 µL of Freund’s complete adjuvant at a ratio of 1:1 (*v*/*v*). Two weeks after the first immunization, the rabbits were boosted with four additional subcutaneous injections with 400 µg of the purified protein mixed with 400 µL of Freund’s incomplete adjuvant per injection at a ratio of 1:1 every week. At 35 days post-injection (dpi), blood samples from the auricular vein of the rabbits were harvested for the titer evaluation assay.

The titer of the antiserum against AcCRaV-CP proteins was determined via indirect ELISA method. Recombinant protein (1 µg/mL) was used. When the titer was greater than 1:50,000, blood samples were taken to prepare the antiserum. Whole blood was collected, and the crude antiserum was obtained via centrifugation. The crude polyclonal antibody was purified with a protein A spin kit (Thermo Fisher Scientific, San Jose, CA, USA) according to the manufacturer’s instructions. The titer of the purified antibody was detected via indirect ELISA, and the concentration of the obtained antibody was resolved to utilize a BCA protein concentration determination kit (Thermo Fisher Scientific, San Jose, CA, USA). The purity of the purified antibody was visualized via SDS-PAGE and Coomassie blue staining. The resulting PAb-AcCRaV-CP was combined with glycerol and sodium azide to final concentrations of 50% and 0.1%, respectively. The purified PAb-AcCRaV-CP was conjugated with HRP at a mass ratio of 1:1. The HRP labeling PAb-AcCRaV-CP (PAb-AcCRaV-CP-HRP) was detected for titer via indirect ELISA [17]. HRP-conjugated Goat Anti-Rabbit IgG (Shenggong Biological Co., Ltd., Shanghai, China) was used in this study.

### 2.5. Potency Detection of Polyclonal Antibodies

The titer of the AcCRaV-CP polyclonal antibody was determined using western blot and DAS-ELISA (the detailed ELISA testing procedures are shown in Section 2.6). The bacterially expressed recombinant protein was used as the antigen. The antigen was diluted to 1 µg/mL with PBS, and a 96-well microplate was prepared by adding 100 µL of the antigen to each well. A blank control was set up with 1% BSA. Following incubation, the wells were washed three times with washing buffer (PBS containing 0.05% Tween 20) for 5 min each to remove unbound antigen. Next, 100 μL of blocking buffer (1% BSA in PBST) was added to each well, and the plate was incubated at 37 °C for 2 h. The plate was then washed as previously described. Several dilutions of the primary antibody in PBST, including 1:500, 1:1000, 1:2000, 1:5000, 1:8000, 1:10,000, 1:20,000, and 1:102,400, were prepared, and 100 μL of each dilution was added to the wells. The plate was incubated at 37 °C for 2 h, followed by washing as explained. Next, 100 μL of HRP-labeled goat anti-rabbit IgG, diluted 1:5000 in PBST, was added to each well, and the plate was incubated at 37 °C for 1 h. The wells were subsequently washed three times as described to remove any unbound secondary antibodies. Finally, 100 μL of TMB substrate solution was added to each well, and the plate was incubated at 37 °C in the dark for 15–30 min. Absorbance was measured at 450 nm using the Microplate Reader Model 680 (BIO-RAD, Hercules, CA, USA). The reaction was considered positive when the mean OD values were at least 2.1 times higher than those of the blank control. The reaction was terminated by adding 100 μL of 2 mol/L H_2_SO_4_ to each well, changing the color from blue to yellow [17].

An amount of 40 μL purified recombinant protein was employed for western blot analysis. Firstly, SDS-PAGE electrophoresis was conducted, the gel was removed, and subsequently, the membrane was transferred for antibody incubation and imaging.

### 2.6. Establishment of the ELISA Detection System and RNA Extraction

To prepare samples for ELISA specificity validation, one gram of plant tissue leaves infected with AcCRaV, SMV, Cucumber mosaic virus (CMV, Cucumovirus CMV) from the virus genus Cucumovirus, AcVA, and AcVB were respectively weighed and grounded into powder with liquid nitrogen. Then, 2 mL of PBST was added at 4 °C, centrifuged at 12,000 rpm for 10 min, and the supernatant was taken as the samples to be tested. Purified recombinant protein and PBST buffer were used as the positive and negative controls, respectively.

To prepare samples for ELISA sensitivity verification, 0.2 g of AcCRaV-infected kiwi leaves were weighed and ground into a powder using liquid nitrogen. Then, 400 μL of PBST was added, and the mixture was further ground to obtain a homogeneous sap. The mixture was centrifuged at 12,000 rpm for 10 min at 4 °C, and the supernatant was collected. The supernatant was then diluted with PBST at ratios of 1:10, 1:50, 1:100, 1:200, 1:500, 1:800, 1:1000, and 1:2000 to prepare a series of samples for subsequent or downstream testing.

To prepare samples for ELISA testing of the field, 1 g of kiwifruit leaves with suspected virus were collected from the field and were weighed. The total RNA from the leaves was extracted, and RT-PCR detection was performed. The primer sequence used is shown in Supplementary Table S1. One gram of plant tissue leaves infected with AcCRaV (*Emaravirus actinidiae*) was weighed and ground into a powder with liquid nitrogen. Then, 2 mL of PBST was added to the powder. It was centrifuged for 10 min at 4 °C and 12,000 rpm, and the supernatant was taken as the sample to be tested. Purified recombinant protein and healthy kiwi leaves were used as the positive and negative controls, respectively.

The ELISA test steps were as follows. Detection of AcCRaV in plant tissues was then performed following the procedure described by Li et al. [18], with slight modifications. Briefly, 1 g of kiwifruit leaf tissues was ground in liquid nitrogen and then homogenized in 2 mL of 0.01 mol·L^−1^ phosphate buffered saline (PBS, pH 7.4). The extract was centrifuged for 5 min at 8000× *g* and the resulting supernatant loaded into wells (100 μL/well) of ELISA microplates followed by 2 h of incubation at 37 °C or overnight at 4 °C. Wells containing crude extracts from the healthy (negative) or AcCRaV-infected (positive) kiwifruit tissues were used as the controls. After 2 h of blocking with a 0.01 PBS (pH 7.4) containing 5% dried skimmed milk, each well was incubated with a diluted polyclonal antibody for 2 h at 37 °C followed by incubation for 1 h with the HRP-conjugated Goat Anti-Rabbit IgG (Shenggong Bioengineering (Shanghai, China) Co., Ltd.) at 37 °C. The wells were washed 3–4 times with PBS containing 0.05% tween-20 (PBST) between the different steps. Then, we used the TMB color reagent kit (Vazyme Biotech Co., Ltd., Nanjing, China) to develop the color according to the manufacturer’s instructions. After terminating the reaction, the absorbance at OD_450_ was measured with a Microplate Reader Model 680 (BIO-RAD, Hercules, CA, USA). A sample was considered to be positive when its absorbance value was at least 2.1 times greater than that of the negative controls. A total of 10 AcCRaV-negative samples (healthy kiwifruit leaf tissue) were tested with DAS-ELISA. The critical value was calculated using the following relation: Χ + 3SD, where Χ represents the mean value of OD_450_ of the 10 negative samples, and 3 SD represents 3 standard deviations [19].

### 2.7. Establishment of the DIBA Detection System

Kiwi leaves infected with AcCRaV (0.2 g) were weighed and ground to a powder with liquid nitrogen. Then, 400 μL of PBST was added, and centrifugation at 4 °C and 12,000 rpm was carried out for 10 min. The supernatant was removed, and the mixture was diluted with PBST 0, 10×, 50×, 100×, 200×, 500×, 800×, 1000×, and 2000×, as the samples to be tested, setting a positive control and negative control. The nitrocellulose membrane (NC membrane) was cut into the appropriate size. A 10 mm × 10 mm lattice was marked on the membrane using a pencil. Then, 10 µL of the infected leaf grinding liquid was deposited onto the NC film and allowed to dry at room temperature for 20 min. Then, 20 mL of 5% nonfat dry milk blocking solution diluted in PBST was added to the dish, and it was blocked for 90 min at room temperature. Then, 10 mL of 1:5000 primary antibody solution diluted in PBST was added and incubated at room temperature for 1 h and 30 min. The primary antibody solution was withdrawn, and the membrane was washed three times with PBST for 5 min each time. The washing solution was poured out, and 10 mL of 1:5000 HRP labeled sheep anti-rabbit IgG solution diluted in PBST was added and incubated for 90 min at room temperature. The secondary antibody solution was withdrawn and washed three times with PBST for 5 min each time. The NC membrane was removed and placed into TMB chemosorption in the dark for 10–15 min. The membrane was washed with distilled water, and the reaction was terminated [20].

Fresh kiwi samples with AcCRaV, SMV, CMV, AcVA and AcVB were screened using RT-PCR (the primers used are listed in Supplementary Table S1). The kiwi leaf samples were collected from Sichuan during spring. One gram of kiwi leaves was ground into a fine powder using a mortar and pestle with liquid nitrogen. Subsequently, 3 mL of PBST was added, and the mixture was further homogenized. The resulting sap was centrifuged at 12,000 rpm for 10 min at 4 °C. A 200 μL aliquot of the supernatant was then collected and subjected to DIBA (as described in the first paragraph of Section 2.7). Purified protein and PBST buffer were used as the positive control and negative control.

### 2.8. Preparation of a Colloidal Gold-Conjugated Polyclonal Antibody

The antiparticle morphology of the precipitated colloidal gold solution was observed under a transmission electron microscope (transmission electron microscope, (TEM), JEM-2100Plus, Tokyo, Japan). The optimal concentration and pH of gold-standard antibodies were established through screening. We took a clean beaker and utilized 0.2 mol/L of K_2_CO_3_, adjusted to the optimal pH value. Then, we took an appropriate amount of polyclonal antibody to the colloidal gold solution of pH and slowly added it drop by drop, while stirring at room temperature for 1 h. Then, 10% BSA was added to a final concentration of 1%, and it was stirred at room temperature for 30 min. This was later split into a 2 mL centrifuge tube and centrifuged at 4 °C and 2000 rpm for 20 min to discard the precipitate formed by the condensed gold particles. The red supernatant was removed and transferred to a fresh centrifuge tube at 4 °C and 8000 rpm for 30 min, and the supernatant was discarded. Resuspension in 1% BSA was repeated twice at 4 °C and 8000 rpm for 20 min, and the supernatant was discarded. The dark red precipitate from the flow at the bottom of the tube was the gold standard antibody. The dark red precipitate from the flow at the bottom of the tube was collected, re suspended with 2 mL 1% BSA, and stored at 4 °C away from light. The resuspension formulation optimization is presented in Supplementary Table S2.

### 2.9. Development and Test Procedure of the Immunochromatographic Strips

The immunochromatographic strip is composed of a sample pad, a conjugate pad, a nitrocellulose (NC) membrane, and an absorbent pad. Polyester membranes, absorbent papers, sticky bases and plastic cases were purchased from Shanghai Jinbiao Biotechnology Co., Ltd., Shanghai, China.

The glass fiber membrane SB 08 (Shanghai Jinbiao Biotechnology Co., Ltd.) was cut into a size of 4 mm by 6 mm. First, the blocking solution (4% BSA) was placed for sealing for 2 h and then removed. Then, it was baked for 1 h 20 min at 42 °C. Several cut and treated glass fiber films were immersed into purified gold-standard antibody solution and kept at 4 °C overnight. The glass fiber film was removed, put in a 42 °C oven, and dried for 80 min. It was placed in a container containing desiccant (anhydrous calcium chloride) and kept at 4 °C away from light. The NC membrane was precisely cut to the dimensions of 4 mm by 23 mm, with 0.6 µL of primary antibody (1 mg/mL) applied at a distance of 7 mm from the left edge as the detection line (T line), and 0.6 µL of sheep anti-rabbit IgG (1 mg/mL) was positioned at 15 mm for the quality control line (C line). After drying at room temperature, the NC film was stopped in blocking solution (1% BSA) for 2 h. Then, it was removed, dried at room temperature, and placed in a container with desiccant and stored for 4 °C. The glass fiber membrane SB 08 was cut to 4 mm by 20 mm, sealed with PBST for 2 h, put in a 55 °C oven and dried for 3 h for later use. The rubber board was cropped to a size of 4 mm by 60 mm. The sample pad, gold label pad, NC film, and absorbent paper were pasted on the rubber board from left to right. The gold-standard pad was under the sample pad, with an overlap of 3 mm between the two, and pressed. The NC film was pasted under the gold pad. One side of the NC film T line was on the brink of the gold pad. The two overlapped by 3 mm. Absorbent paper was pasted on the other side of the NC film C line, which was higher than the NC film, overlapping by 3 mm and was pressed. The strip was placed into the casing [17].

### 2.10. Test of Colloidal Gold Immune Analysis Test Strip

Sensitivity analysis: the bacterial solution containing AcCRaV-CP recombinant protein was taken as the antigen, then diluted 10×, 50×, 100×, 200×, 500×, 1000×, and 2000×. Utilizing the colloidal gold immunochromatography strip, 200 µL of lysate was dispensed onto the sample pad and allowed to incubate for 5 to 10 min.

Specificity analysis: Leaves infected with AcCRaV, SMV, CMV, AcVA, and AcVB were weighed to 1 g, put into a sterile mortar, and ground to a powder with liquid nitrogen. Then, 2 mL of PBST was added, and centrifugation was carried out at 4 °C and 12,000 rpm for 10 min. The supernatant was taken as the sample to be tested, and 1 PBST buffer was used as the negative control. Using the colloidal gold immunochromatography strip, a 200 µL sample was taken to be tested on the sample pad and left to sit flat for 5–10 min to observe the results.

### 2.11. Detection of the Field Samples via Colloidal Gold Immune Analysis Test Strip

Leaves from the field were weighed to 1 g, put in a sterile mortar, and fully ground into a powder with liquid nitrogen. Then, 3 mL of PBST was added, and centrifugation was carried out at 4 °C and 12,000 rpm for 10 min. The supernatant was taken as a sample to be tested, and healthy kiwi leaves were used as a negative control. Then, 200 µL of the supernatant was placed on the sample pad and left to sit flat for 5–10 min to observe the results.

### 2.12. Statistical Analysis

The one-way ANOVA model was used for analyses of the error in IBM SPSS Statistic 27, and the average value was obtained. The significance was assessed using the new complex range method (Duncan’s method) at *p* < 0.01. The relative concentrations of a 1:1 dilution of the antigen were comparably similar between all assays.

## 3. Results

### 3.1. Analysis of the AcCRaV-CP Sequence Clustering and Cloning

**Figure 1:**
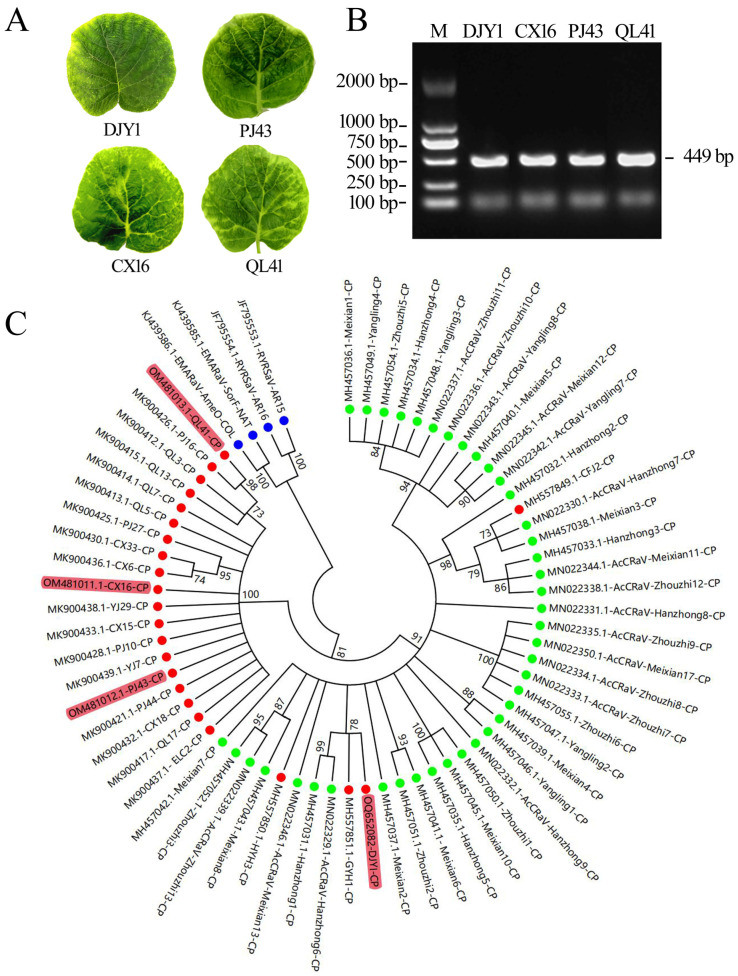
Identification of the four AcCRaV strains and sequence analysis. (**A**) Leaf symptoms of AcCRaV infection. (**B**) Identification of AcCRaV by RT-PCR. (**C**) sequence analysis based on AcCRaV-CP.

The genetic variation and geographical distribution of the nucleoprotein gene sequence exhibited a positive correlation. We successfully amplified the nucleoprotein gene sequence from four samples collected in Pujiang, Dujiangyan, Cangxi, and Qionglai. These infected leaves all showed distinct mosaic symptoms (Figure 1A). The total RNA was extracted from symptomatic kiwifruit leaves and the cDNA was reverse-transcribed. Using the above cDNA as template, PCR amplification was performed with AcCRaV specific detection primers (Supplementary Table S1) to obtain a 499 bp amplification product (Figure 1B). Subsequently, cluster analysis was performed on these four sequences along with the published *AcCRaV-CP* sequences. The AcCRaV-CP-DJYI, AcCRaV-CP-QL41, AcCRaV-CP-CX16, and AcCRaV-PJ43 sequences in this study (marked in red in the figure) and the NCBI reference sequences were analyzed using Muscle of MEGA7.0 software. Isolates of the European mountain ash ringspot-associated virus (EMARaV) (AMO-COL and SorF-NAT) and Redbud yellow ringspot-associated virus (RYRSaV) (AR15 and AR16) were used as outgroups. The reference sequences were 41 kiwifruit isolates reported from Shaanxi Province [3] and 18 AcCRaV-CP sequences previously reported by us [2]. The neighbor-joining method (NJ) was adopted for analysis. The phylogenetic data showed that there was nearly a 1–6% variation in the nucleic acid sequences of the nucleoprotein genes of the strains from different regions, and the similarity of the encoded proteins was 99.00–100.00%. The sequences from Shaanxi Province were roughly clustered together, and the sequences from Sichuan Province were roughly clustered together. It can be seen that the nucleoprotein gene sequence evolution of AcCRaV was affected by geographical location. The consensus sequences of the nucleoprotein gene were used as a foundation for subsequent antibody design.

**Figure 2:**
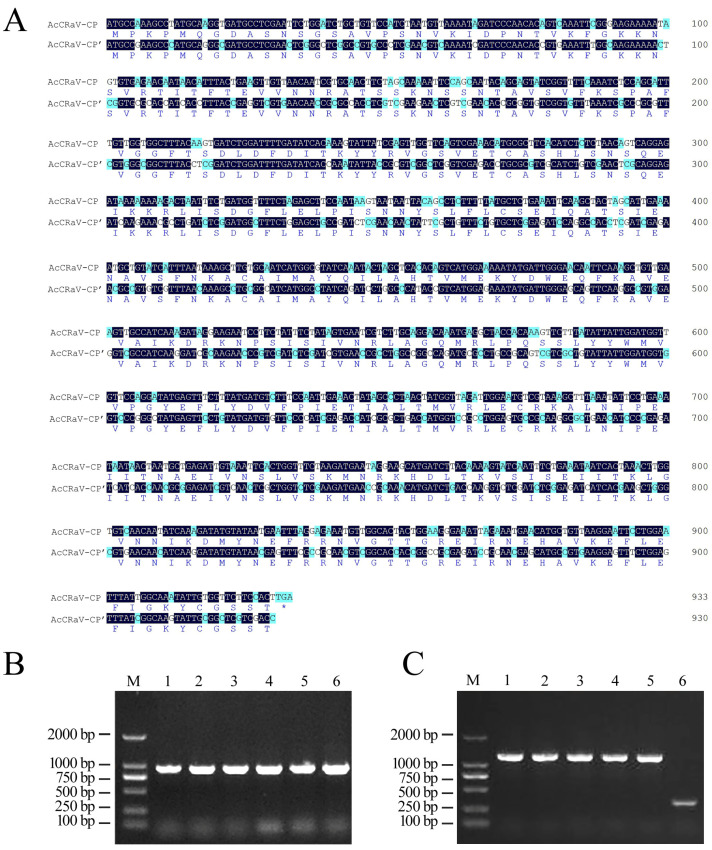
Comparison of sequence optimization results of AcCRaV-CP gene and expression vector constructs. (**A**) Comparison of sequence optimization results of AcCRaV-CP gene. (**B**) AcCRaV-CP gene amplification with restriction sites; Lanes 1–6 were repeated to show the products of amplification of AcCRaV-CP target gene. (**C**) PCR detection based on the monoclones of AcCRaV-CP gene; Lanes 1–5 were repeated to show recombinant plasmid positive clones. Lane 6 is empty plasmid pET28a.

We utilized the nucleoprotein gene sequence of isolate DJY 1 as a template to extend the entire coding region of the nucleoprotein (Figure 2A). The resulting AcCRaV-CP gene sequence, AcCRaV-CP-CDS, was subjected to codon optimization in order to obtain the AcCRaV-CP’ gene sequence, while maintaining an unchanged protein sequence (Figure 2B). The amplified product using the AcCRaV-CP’ gene sequence as a template was provided by enzyme digestion and ligation, resulting in the recombinant plasmid pET28a-AcCRaV-CP-DJY1. This recombinant plasmid was kept in *E. coli* DH5α for bacterial liquid PCR analysis (Figure 2C), which confirmed the correct sequencing.

### 3.2. Induced Expression of AcCRaV-CP Protein

**Figure 3:**
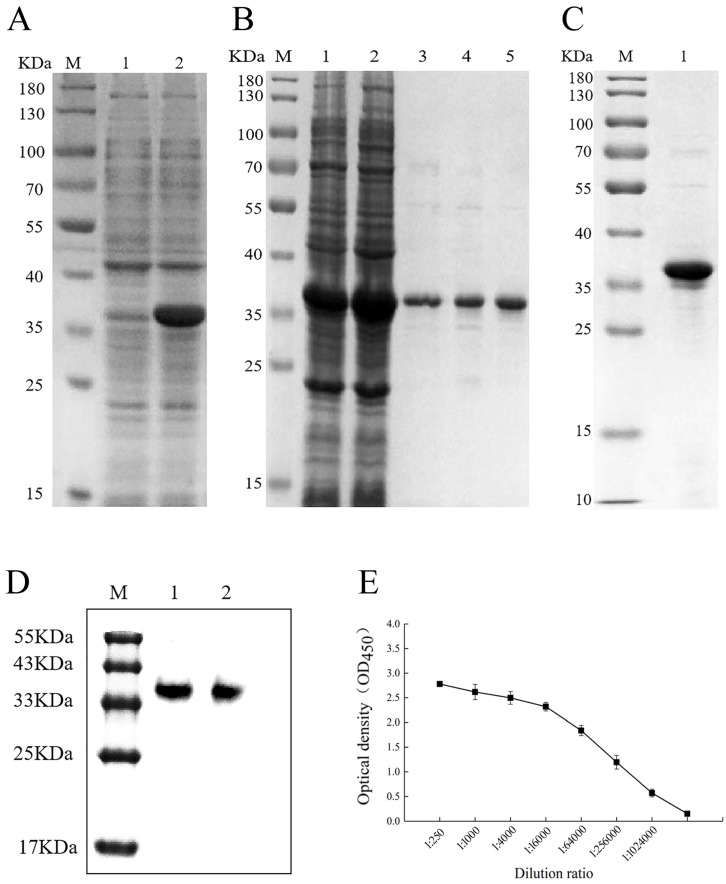
Expression and purification of the recombinant protein. (**A**) The expression of AcCRaV-CP was determined via sodium dodecyl sulfate–polyacrylamide gel (SDS-PAGE) analysis; Note: lane M: marker, Lane 1: without inducer, Lane 2: final concentration of IPTG 1 mmol/L. (**B**) The purification of AcCRaV-CP was determined via SDS-PAGE; Note: Lane M: marker, Lane 1: after crushing, Lane 2: effluents, Lanes 3–5: eluent. (**C**) Recombinant protein was determined via SDS-PAGE; Note: Lane M: marker, Lane 1: dialysis sample. (**D**) Specificity of the AcCRaV-CP polyclonal antibody was determined via western blot; Note: Lane M: marker, Lane 1–2: western blotting. (**E**) Detection of OD450 value of AcCRaV-CP polyclonal antibody titer via ELISA.

The first induction of the recombinant plasmid pET28a-AcCRaV-CP-DJY1 in the Rosetta (DE3) strain resulted in a protein with a molecular weight of 35.4 KDa (Figure 3A). Subsequently, the recombinant plasmid was second induced for a second time. The recombinant protein was purified using Ni-NTA Resin, as depicted in Figure 3B. The elution sample strips in Columns 3 to 5 were single, and the molecular weight of 35.4 KDa is correct, suggesting that the recombinant protein had been successfully purified and could be utilized for subsequent dialysis experiments (Figure 3B). The recombinant protein successfully purified with Ni-NTA Resin was transferred into a dialysis bag and dialysis was performed with 0.01 mol/L PBS. The SDS-PAGE test results are shown in Figure 3C.

### 3.3. Potency Detection of Polyclonal Antibody

The AcCRaV-CP polyclonal antibody was diluted to a concentration of 1:10,000. Then, 5 µL of protein was used for SDS-PAGE, and the concentration of the used gel(s) was 12.5%. HRP-conjugated goat anti-rabbit IgG was used in this study. The purified protein exhibited specific immunoreactivity as evidenced by a distinct single band, indicating the high specificity of the antibody toward AcCRaV-CP protein (Figure 3D). Subsequently, ELISA was performed using dilutions of the AcCRaV-CP polyclonal antibody at 1:250, 1:100, and 1:4,000,000. The optical density (OD) values were measured at 450 nm using a microplate reader, and the average value was calculated from three replicates. The P/N value was established by dividing each sample’s OD_450_ average (P) by 2.1 times the OD_450_ value of the blank control sample (N). A positive result indicated that P exceeded N by 2.1 times; thus, P/2.1N > 1 denoted positivity, while results below this threshold were considered negative. Based on these calculations, both antibodies displayed titers above 1:1,024,000, in accordance with the ELISA results, confirming their high titer and excellent quality (Figure 3E).

### 3.4. Establishment of ELISA for AcCRaV

**Figure 4:**
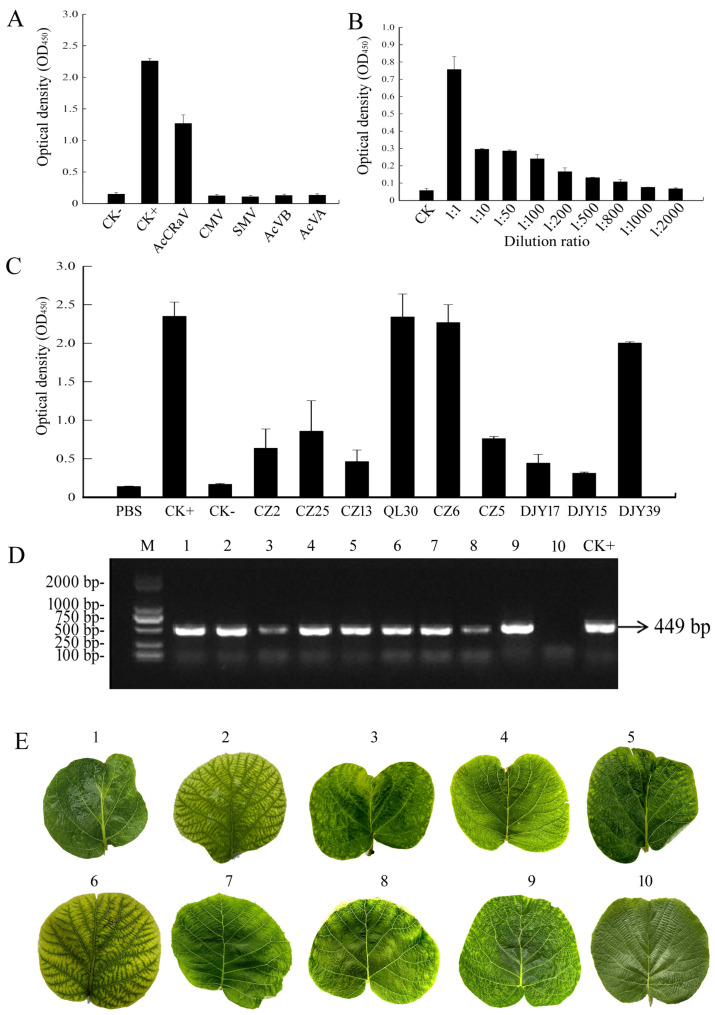
Application of the ELISA. (**A**) Specificity detection of ELISA. (**B**) Sensitivity detection of the ELISA. (**C**) Field samples were detected by ELISA. (**D**) Field samples were determined by RT-PCR. (**E**) Symptoms of the field samples. Note: 1: CZ 2, 2: CZ 25, 3: CZ 13, 4: QL 30, 5: CZ 6, 6: CZ 5, 7: DJY 17, 8: DJY 15, 9: DJY 39, 10: healthy leaf, set as CK−, CK+: positive control infected with virus.

The AcCRaV specificity was determined using ELISA. Sheep anti-rabbit IgG labeled with ACCRAV-CP polyclonal antibody and HRP was diluted to a working concentration of 1:500. Following the ELISA procedure, homogenates from plants infected with AcCRaV, SMV, CMV, AcVA, and AcVB were utilized. The OD_450_ results indicate positive for AcCRaV and negative for the other four viruses (SMV, CMV, AcVA, and AcVB), demonstrating high specificity of the ELISA detection in distinguishing AcCRaV from these viruses. The ELISA specificity test results demonstrate the high detection specificity of this method, enabling effective discrimination between AcCRaV and the tested viruses (Figure 4A).

Moreover, the sensitivity of the ACCRAV-CP antibody was assessed using ELISA. The first polyclonal anti-AccRAV-CP antibody and the second HRP-labeled sheep anti-rabbit IgG were diluted to a working concentration of 1:500. Kiwi samples infected with AcCRaV were subjected to abrasion at dilutions of 10, 50, 100, 200, 500, and 800 times. The results from the dilutions of 1000 and 2000 times showed that the ELISA method could detect kiwi fruit diluted to 500 times AcCRaV with a positive result; however, the dilutions greater than 500 times yielded negative results. The ELISA sensitivity results revealed its capability to detect kiwi AcCRaV even when diluted up to 500 times, yielding positive signals while remaining negative beyond this dilution threshold (Figure 4B).

The leaves collected from the field were subjected to ELISA analysis. CZ2, CZ25, CZ13, QL30, CZ6, CZ5, DJY17, DJY15, and DJY39 produced positive results while DJY35 and DJIY33 tested negative (Figure 4C). These findings are in line with the outcomes of the RT-PCR assay (Figure 4D), indicating that ELISA exhibits high accuracy and can be effectively employed for large-scale testing in field conditions. Notably, all field samples exhibited symptoms of fading (Figure 4E).

### 3.5. Establishment of the Spot Immunization Method

**Figure 5:**
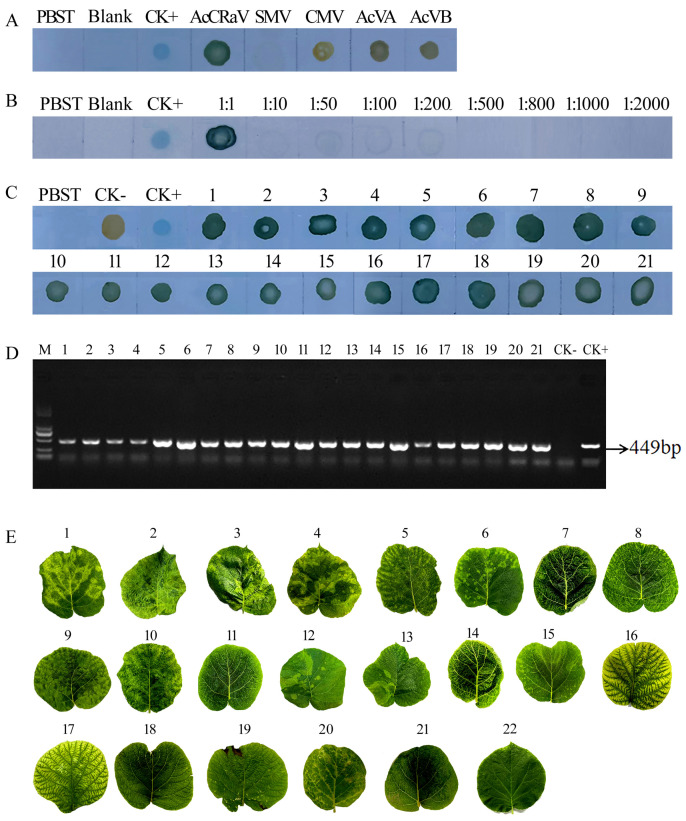
Application of the DIBA assay. (**A**) Specific detection of the DIBA assay. (**B**) Sensitivity detection of DIBA assay. (**C**) Field samples were detected by DIBA assay. (**D**) Field samples were determined by RT-PCR. (**E**) Symptoms of the field samples. Note: 1: JD63, 2: JD104, 3: DJY36, 4: JD73, 5: JD79, 6: JD82, 7: JD108,8: DJY39, 9: DJY8, 10: XGZ55, 11: DJY38, 12: XGZ47, 13: JD72, 14: DJY18, 15: DJY25, 16: CZ5, 17: CZ25, 18: QL2, 19: QL3, 20: YA23, 21: YA44, 22: healthy leaf, CK−: healthy leaf, CK+: positive control infected with virus, Blank: a blank control without viral proteins.

The DIBA method demonstrated a positive result for AcCRaV detection, as indicated by the appearance of blue color. In contrast, it yielded negative results for the tested viruses. Sensitivity analysis revealed that the grinding solution did not exhibit any color change due to its extremely low virus particle content (Figure 5A). Furthermore, specificity analysis confirmed the high specificity of the DIBA test method.

The symptomatic leaves collected from the field were subjected to DIBA testing, yielding positive results that are consistent with RT-PCR (Figure 5C,D). This method demonstrates high reliability and suitability for carrying out a large number of tests on field samples. The symptoms observed in kiwi leaves infected with AcCRaV primarily manifested as extensive fading, although a few showed no obvious symptoms (Figure 5E).

### 3.6. Controlled Test and Field Application of the AcCRaV-GICS

**Figure 6:**
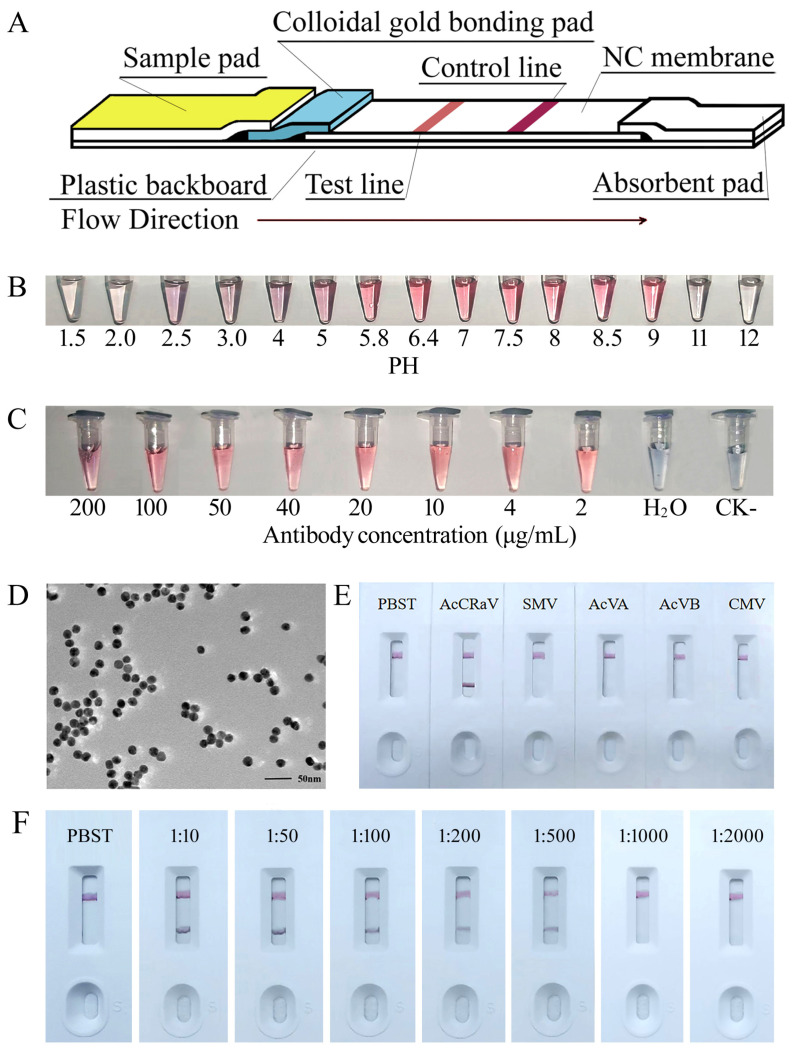
Development, sensitivity and specificity test, and field application of the AcCRaV-GICS. (**A**) The schematic representation of the AcCRaV-GICS; (**B**,**C**) Optimum PH and most applicable amount of colloidal gold-labeled AcCRaV-CP antibody. (**D**) Colloidal gold particles visualized by TEM. (**E**) specificity test of the AcCRaV-GICS in detection of AcCRaV, SMV, AcVA, AcVB, and CMV. (**F**) sensitivity test of the AcCRaV-GICS in detection of AcCRaV present in the gradient-diluted crude extract of leaves infected with AcCRaV.

During the test, a volume of 2.5 mL of colloidal gold solution was added, leading to well-dispersed and uniformly sized gold antiparticles of around 20 nm, as observed under the transmission electron microscope (Figure 6D). The colloidal gold solution exhibited a stable red color within the pH range of 6.4 to 9.0, rendering it suitable for antibody labeling (Figure 6B). Deviations from this pH range can cause the colloidal gold solution to turn purple or gray [18]. At a final concentration of antibody in the colloidal gold solution at 2 μg/mL, the solution remained red and exhibited stability, thus indicating that this concentration is optimal for colloidal gold-labeled antibody application (Figure 6C). In practical labeling procedures, the protein content can be improved by an additional 10% to 20%.

The immunocolloidal gold chromatographic strips (GICS) were assembled as depicted in Figure 6A. The sensitivity detection results demonstrate that the T line gradually faded from deep to shallow with increasing dilution. When the strip was diluted 1000 times, no color was observed on the T line, indicating a failure to detect the antigen. This indicates that the colloidal gold strip can identify a 500-fold dilution containing AcCRaV-CP ultrasonic lysate with a sensitivity of 1:500 (Figure 6E). Specific test results reveal that only leaves infected with AcCRaV exhibited two lines (the T line and C line), indicating positive results. The leaves infected with SMV, AcVA, AcVB, CMV, and negative-control PBST showed only the C line (Figure 6F). These findings highlight the specificity of AcCRaV-GICS.

**Figure 7:**
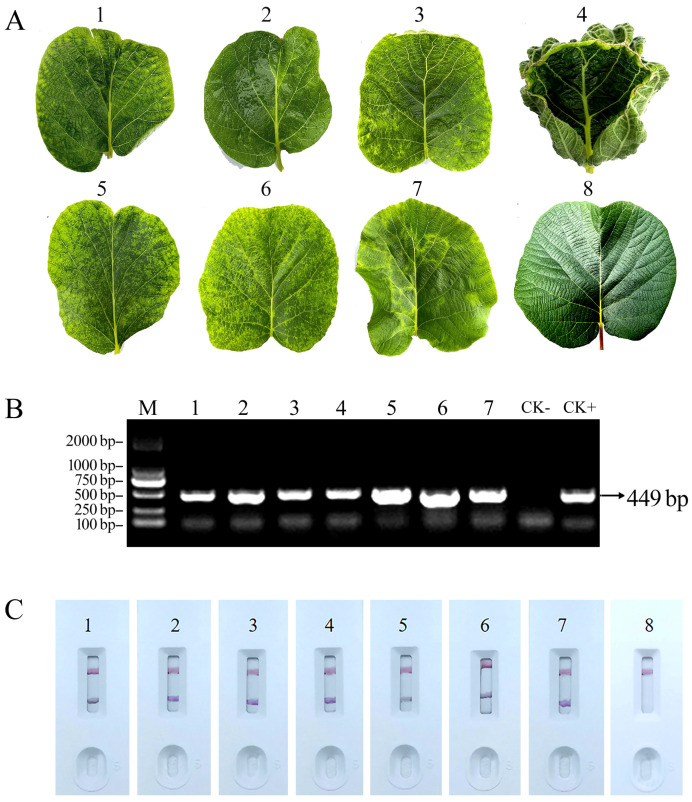
Display of some samples for field application of the AcCRaV-GICS. (**A**) AcCRaV-infected leaves in the field. (**B**) detection of AcCRaV present in the leaves of the field plants using RT-PCR. (**C**) detection of AcCRaV in field samples using the AcCRaV-GICA strip. Note: 1: CZ6, 2: CZ2, 3: JD21, 4: JD80, 5: JD60, 6: XGZ41, 7: JD72, 8: healthy leaf, CK−: healthy leaf, CK+: positive control infected with virus.

Detection of the field samples was performed using a colloidal gold immunochromatography strip (Figure 7). All AcCRaV-positive samples tested using RT-PCR (Figure 7B) exhibited positive results on the test strips as well (Figure 7C). The colloidal gold immunochromatography strip method offers a simple and rapid detection approach, providing results within 6–10 min.

**Figure 8:**
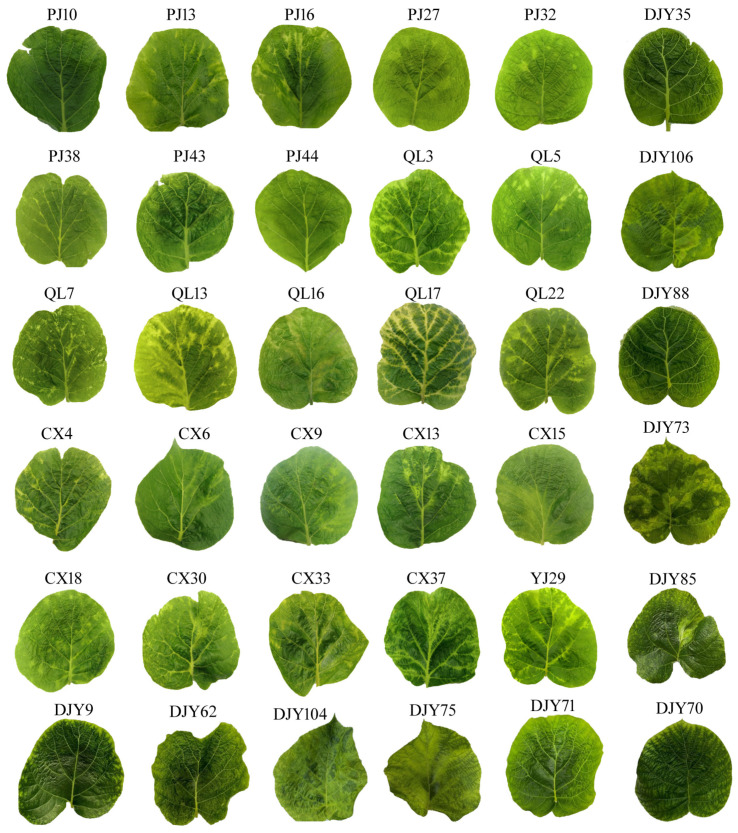
Leaf symptoms that were tested positive after AcCRaV-GICS in a large number of field samples.

To further demonstrate AcCRaV-GICS in detecting field samples, we conducted extensive testing on a diverse range of samples collected from various regions. The detection results obtained are considered to be consistent with the RT-PCR results (Supplementary Table S3). A majority of the definite leaves exhibited significant areas of chlorosis and mosaic symptoms, while some leaves displayed deformities (Figure 8). Our previous investigations have demonstrated that the manifestations of viral diseases in kiwifruit are intricate, particularly in field samples. It is impossible to determine the type of virus in a single or mixed infection based on the symptoms alone. Nevertheless, we discovered that the majority of mixed infections would exhibit more severe symptoms compared to samples with a single infection [2].

## 4. Discussion

The detection rate of AcCRaV was the highest in the Sichuan kiwi leaves. Its incidence is proportional to tree age and inversely proportional to altitude [2]. With the continuous expansion of kiwifruit planting area and the increase in trade, good conditions have been created for the spread of the virus. Viral diseases lack effective control agents and can only be stopped when diagnosed in time and stopped from spreading. Therefore, it is crucial to establish a fast and accurate detection method.

Considering the complexity of kiwi virus disease symptoms, we urgently require a method capable of accurately identifying the kiwi virus. The AcCRaV-CP polyclonal antibody was employed in this study to develop three detection methods: DAS-ELISA, spot immunization (DIBA), and colloidal gold immunochromatographic strip (GICS) for AcCRaV.

The detection of AcCRaV using ELISA and DIBA demonstrated accurate results. Notably, the DIBA method required the lowest number of samples for detection. The extraction process presents significant challenges owing to the elevated polysaccharide content found in kiwi leaves [21]. Employing a crude virus extract as a coated antigen greatly simplifies the detection procedure. In order to avoid interference from brown blotting caused by the rapid oxidation of phenols in kiwi tissue when dripping grinding droplets of symptomatic leaves onto NC membranes during the DIBA test, we opted for 3,3′,5,5′-tetramethylbenzidine (TMB) chromogen as the chromogenic substrate. TMB can be converted into blue under peroxidase catalysis, facilitating easy observation.

By utilizing a colloidal gold immunochromatography strip, AcCRaV can be detected within 6 to 10 min. The strip exhibits high specificity toward AcCRaV and the results obtained from the dipstick test are in agreement with those from PCR analysis. Colloidal gold immunochromatography test strip (GICS) possess the advantages of simplicity, rapidity, and low technological requirements [22]. This method is well-suited for the efficient detection of a large number of samples in field settings, thereby significantly facilitating AcCRaV detection and prevention.

Preparation of the test strip requires pristine experimental equipment. The experimental water utilized was ultra-pure, double-distilled water with a resistivity of 18.2 megohm or above. It was noted that the colloidal solution presented a wine-red color, remarkable clarity, no turbidity or debris, and a uniform particle size distribution, making it appropriate for labeling antibodies with colloidal gold. The quality of the colloidal gold solution was affected by various factors such as the quality of raw materials like chloroauric acid, the amount of reducing agent employed, reaction time, stirring speed, and others [17]. During the preparation process of the colloidal gold solution, trisodium citrate should be added rapidly and stirred vigorously [23].

In this study, the pore size of the NC membrane, the antibody resuspension, gold pad, and sample pad were optimized. A smaller pore size was selected for the NC membrane to enhance its sensitivity. Furthermore, the optimal concentration for antibody labeling was determined. The gold standard antibody resuspension formula was employed, leading to a dense and dark purified gold standard antibody without any aggregation phenomenon, indicating excellent quality. To enhance performance, the gold label pad was subjected to pretreatment with a sealing solution comprising 4% BSA, and the sealing duration was extended to 2 h. Likewise, the sample pads were pre-treated with PBST and the coating time was extended to 2 h. Additionally, cells were blocked with 1% BSA for 2 h. These optimization treatments significantly decreased false negatives and false positives.

Three serologic methods, namely double-antibody sandwich enzyme-linked immunoassay (DAS-ELISA), dot immunoassay (DIBA), and colloidal gold immunochromatographic strip (GICA), for the rapid detection of AcCRaV were established for the first time in this study. The traditional RT-PCR detection method requires technical skills and specific instruments, and it is time-consuming and not suitable for large-scale sample detection. The three serological approaches can detect a considerable number of field samples with high efficiency and sensitivity. DAS-ELISA tests can be both qualitative and quantitative, requiring specific apparatuses and professional operations. DIBA detection can only be performed through qualitative analysis. If the virus content is low, it is challenging to capture color. Due to the particularity of polyphenol content in kiwifruit leaves, eliminating the interference of its background color is of utmost importance for the determination of DIBA results, so it is highly significant to select the appropriate color-developing substrate. When using a colloidal gold immunochromatographic test strip to detect AcCRaV, without the need for special instruments and skills, it is light and efficient, with the results observable within 6 to 10 min, showing great potential for commercial development. Our previous studies indicated that AcCRaV was frequently combined with AcVA, AcVB, CMV, and other viruses to infect kiwi trees [2]. The development of double- or multiple-compound colloidal gold immunochromatographic strips capable of simultaneously detecting multiple kiwi viruses is one of the future research directions. Since the immunochromatographic test strip in this experiment was purely handmade, the colloidal gold test strips among different batches were not particularly aesthetically pleasing in appearance and there was certain instability in performance. If optimized with the assistance of some instruments and equipment, such as using a film-spraying machine for film scratching and a cutting machine for material cutting, the test strips will be more aesthetically appealing in appearance and more stable in performance. Additionally, for colloidal gold immunochromatography strips, the quality of antibodies is the most crucial factor influencing their effect, so there is still some scope for optimization in the research on colloidal gold immunochromatography strips for the detection of AcCRaV.

## 5. Conclusions

The antigens utilized in this project were derived from the nucleoprotein of AcCRaV. The codon optimization method was employed to optimize the nucleoprotein genes, construct novel polyclonal antibodies, and establish various serological methods, including ELISA, dot immuno-binding assay, and AcCRaV-specific gold immunochromatography strip (AcCRaV-GICS). Its high specificity and sensitivity were verified in field samples. Compared to RT-PCR, RT-LAMP, DAS-ELISA, and DIBA, the colloidal gold immunochromatography strip is simpler and more suitable for the rapid detection of large samples in the field. The development of this detection system will provide an important reference for the early diagnosis of AcCRaV.

## 6. Patents

There are patents resulting from the work reported in this manuscript. The patent relates to the preparation of polyclonal antibodies to Actinidia chlorotic ringspot-associated virus.

## Data Availability

Data are contained within the article and Supplementary Materials.

## Supplementary Materials

The following supporting information can be downloaded at: https://www.mdpi.com/article/10.3390/v16101600/s1, Table S1: Primers used in experiments; Table S2: The resuspension formulation optimization; Table S3: Large numbers of field samples were tested by AcCRaV-GICS and RT-PCR.
