# Supplementary material for: Preparation and Application of Polyclonal Antibodies for the Rapid Detection of Actinidia Chlorotic Ringspot-Associated Virus
Source: Viruses. 2024 Oct 11;16(10):1600. doi: 10.3390/v16101600 (PMC11512300; doi:10.3390/v16101600)
Supplement: Supplementary file 1 [file viruses-16-01600-s001.zip › viruses-3147064-supplementary.pdf]

Table S1. Primers used in experiments.

Gene	Primer (5'-3')	Product Size	Genetic Locus	Reference
AcVA-F-471	CATGGCAAAGAATATCTCAAG	471 bp	Actinidia virus A RNA-binding protein (ORF5) gene	Zhao et al. 2018
AcVA-R-471	AGATCCAACCCAGAGTTGAAA			
AcVB-F-342	GTTTGCGAGGAGACGTAGGGC	342 bp	Actinidia virus B RNA-binding protein gene	Blouin et al. 2012
AcVB-R-342	AGTTAAGTGCTCTYGGRGGTGTG			
AcCRaV-F-449	GCTTGCAAGATGTCGATGCAG	449 bp	Actinidia chlorotic ringspot-associated virus isolate ZZ30-5 segment RNA5, complete sequence (727 bp to 1175 bp)	This study
AcCRaV-R-449	TGCAGGGTCTGCTGCTTATTA			
CMV-F-870	GGATGCTTCTCCRCGAGDT	870 bp	Cucumber mosaic virus coat protein gene	Tang et al. 2018
CMV-R-870	GCTGGATGGACAACCCGTTC			
SMV-F-550	AGCTCGCTTCGTCTGGAAAA	550 bp	Soybean mosaic virus coat protein gene	Zhang et al. 2020
SMV-R-550	ATCATCACCCACACGCCATT			
AcCRaV-CP-F-1133	AACTACTTCATAGTAAATCTTACAA	1133 bp	Actinidia chlorotic ringspot associated virus isolate HN-6 segment RNA3 nucleocapsid protein gene(546 bp to 1678 bp)	This study
AcCRaV-CP-R-1133	AGTAGTGAACTCCCATTAATATC	1133 bp		
AcCRaV-CP-F-933	TTAAGTGGAAGAACCACAATAT	933 bp	Actinidia chlorotic ringspot-associated virus isolate ZZ25-6 segment RNA3, complete sequence(717 bp to 1649 bp)	This study
AcCRaV-CP-R-933	ATGCCAAAGCCTATGCAAG	933 bp		
pET-28a-AcCRaV-CP-F	CGGGATCCATGCCGAAGCCCATGCAGG	-		This study
pET-28a-AcCRaV-CP-R	CCCTCGAGGGTCGACGAGCCGCAATACTT	-		
pET28a-T7	TAATACGACTCACTATAGGG	-		This study
pET28a-T7t	GCTAGTTATTGCTCAGCGG	-		

Table S2. The resuspension formulation optimization.

Name	1	2	3	4
BSA(%)	1	—	1	1
saccharose(%)	—	—	5	—
trehalose(%)	—	—	—	1
tween-20(%)	0.05	0.05	0.05	0.05
PBS(1×)	—	+	—	—
PB(0.1M)	—	—	+	—

Note: The + number indicates the addition of the reagent, – which means that the reagent was not added.

Table S3. Large numbers of field samples were tested by AcCRaV-GICS and RT-PCR.

Number	Origin	Latitude and Longitude	Altitude (m)	Varieties	Tree Age	AcCRaV-GICS	RT-PCR
CX13	Cangxi	31.45N, 105.68E	608	Hongyang	7	+	+
CX15	Cangxi	31.45N, 105.70E	608	Hongyang	7	+	+
CX18	Cangxi	31.45N, 105.73E	608	Hongyang	7	+	+
CX28	Cangxi	31.73N, 106.01E	608	Hongyang	7	-	-
CX29	Cangxi	31.73N, 106.01E	608	Hongyang	7	-	-
CX30	Cangxi	31.73N, 106.01E	608	Hongyang	7	+	+
CX33	Cangxi	31.73N, 106.01E	608	Hongyang	7	+	+
CX37	Cangxi	31.73N, 106.01E	608	Hongyang	7	+	+

CX4	Cangxi	31.45N, 105.59E	608	Hongyang	7	+	+
CX9	Cangxi	31.45N, 105.64E	608	Hongyang	7	+	+
CZ20	Chongzhou	30.63N, 103.67E	517	Hongyang	3	-	-
CZ29	Chongzhou	30.63N, 103.67E	517	Hongyang	10	-	-
CZ30	Chongzhou	30.63N, 103.67E	517	Hongyang	10	-	-
CZ35	Chongzhou	30.63N, 103.67E	517	Hongyang	10	-	-
DJY104	Dujiangyan	31.00N, 103.70E	657	Hongyang	10	+	+
DJY106	Dujiangyan	30.96N, 103.83E	657	Hongyang	10	+	+
DJY119	Dujiangyan	31.00N, 103.70E	640	Hongyang	12	+	+
DJY12	Dujiangyan	31.00N, 103.70E	704	Hongyang	12	+	+
DJY128	Dujiangyan	31.00N, 103.70E	640	Hongyang	10	-	-
DJY129	Dujiangyan	31.00N, 103.70E	640	Hongyang	10	-	-
DJY35	Dujiangyan	31.00N, 103.70E	704	Hongyang	12	+	+
DJY62	Dujiangyan	30.96N, 103.83E	657	Hongyang	12	+	+
DJY70	Dujiangyan	30.96N, 103.85E	657	Hongyang	10	+	+
DJY71	Dujiangyan	30.96N, 103.84E	657	Hongyang	10	+	+
DJY73	Dujiangyan	31.00N, 103.70E	657	Hongyang	10	+	+
DJY75	Dujiangyan	30.96N, 103.83E	657	Hongyang	10	+	+
DJY85	Dujiangyan	30.96N, 103.83E	640	Hongyang	12	+	+
DJY88	Dujiangyan	30.96N, 103.83E	657	Hongyang	10	+	+
DJY9	Dujiangyan	31.00N, 103.70E	704	Hongyang	12	+	+
PJ10	Pujiang	30.31N, 103.44E	526	Hongyang	8	+	+
PJ13	Pujiang	30.31N, 103.44E	526	Hongyang	8	+	+
PJ16	Pujiang	30.31N, 103.44E	526	Hongyang	8	+	+
PJ23	Pujiang	30.31N, 103.44E	802	Hongyang	10	-	-
PJ27	Pujiang	30.31N, 103.44E	526	Hongyang	8	+	+
PJ30	Pujiang	30.31N, 103.44E	802	Hongyang	10	-	-
PJ32	Pujiang	30.28N, 103.50E	526	Hongyang	8	+	+
PJ38	Pujiang	30.31N, 103.44E	526	Hongyang	8	+	+
PJ43	Pujiang	30.31N, 103.44E	526	Hongyang	8	+	+
PJ44	Pujiang	30.31N, 103.44E	526	Hongyang	8	+	+
PJ50	Pujiang	30.31N, 103.44E	802	Hongyang	7	-	-
PJ52	Pujiang	30.31N, 103.44E	802	Hongyang	10	-	-
QL12	Qionglai	30.26E, 103.22E	730	Hongyang	16	-	-
QL13	Qionglai	30.46N, 103.46E	530	Hongyang	8	+	+
QL16	Qionglai	30.46N, 103.46E	530	Hongyang	8	+	+
QL17	Qionglai	30.46N, 103.46E	530	Hongyang	8	+	+
QL22	Qionglai	30.46N, 103.46E	530	Hongyang	8	+	+
QL3	Qionglai	30.46N, 103.46E	530	Hongyang	8	+	+
QL5	Qionglai	30.46N, 103.46E	530	Hongyang	8	+	+
QL7	Qionglai	30.46N, 103.46E	530	Hongyang	8	+	+
QL8	Qionglai	30.26E, 103.22E	730	Hongyang	4	-	-
YA28	Yaan	30.20N, 103.24E	810	Hongyang	16	-	-
YA44	Yaan	30.20N, 103.24E	810	Hongyang	7	-	-
YJ29	Yingjing	29.80N, 102.85E	815	Hongyang	3	+	+
